# Rate of testicular histology failure in predicting successful testicular sperm extraction

**DOI:** 10.3389/fendo.2024.1466675

**Published:** 2024-10-10

**Authors:** Stefano Castellano, Francesca Tondo, Ozgur Bulbul, Sabrina Aprea, Emanuela Monti, Edoardo Carnesi, Paolo Emanuele Levi Setti, Elena Albani

**Affiliations:** ^1^ Division of Gynecology and Reproductive Medicine, Department of Gynecology, Fertility Center, Istituti di Ricovero e Cura a Carattere Scientifico (IRCCS) Humanitas Research Hospital, Milan, Italy; ^2^ Department of Biomedical Sciences, Humanitas University, Milan, Italy

**Keywords:** non-obstructive azoospermia, NOA, obstructive azoospermia, OA, testicular biopsy, testicular histology, fertility preservation, cryopreservation

## Abstract

**Background:**

The management of Non-Obstructive (NOA) Azoospermia or Obstructive Azoospermia (OA) patients relies on testicular sperm extraction (TESE) followed by intracytoplasmic sperm injection (ICSI). In NOA patients the sperm recovery is successful in only 50% of cases and therefore the ability to predict those patients with a high probability of achieving a successful sperm retrieval would be a great value in counselling the patient and his partner. Several studies tried to suggest predictors of a positive TESE (e.g. FSH concentration), but most concluded that diagnostic testicular biopsy (histology) is best.

**Methods:**

This is a retrospective analysis of 526 TESE patients. After the extraction of the testis, the resulting sample was immediately given to the embryologist, who examined the tubules for sperm cryopreservation. During the same procedure, a different specimen was destined to the histological analysis. The comparison between the two methodological approaches was carried out through a score.

**Results:**

Concordance between TESE and testicular histology outcomes was found in 70,7% of patients; discordance was found in 29,3% of patients. Among the discordance outcomes, in approximately 95% we found at least 1 sperm in the TESE retrieval, while the histology report did not find any spermatozoa or found not enough compared to our evaluation; in only 5% of cases we did not find any spermatozoa or found not enough compared to what was detected in the testicular histology.

**Conclusion:**

Based on our experience, to increase diagnostic accuracy, a larger biopsy should be sent to the histopathology laboratory; another option may be to use TESE cell suspension (the same embryologists employ for cryopreservation) for cytological evaluation of spermatogenesis.

## 1 Introduction

Azoospermia is defined as the complete absence of spermatozoa upon microscopic evaluation of two centrifuged semen samples (1, 2). It occurs in 1% of men and approximately 10%–15% of the infertile male population, and can be either non-obstructive (NOA) or obstructive azoospermia (OA) depending on the etiology (1).

NOA is caused by testicular failure, which can be primary (testicular) or secondary to hypothalamo-pituitary dysfunction (pre-testicular). OA is caused either by a blockage in the genital tract or ejaculatory dysfunction (post-testicular), such as the absence of the seminal vesicles, as occurs in congenital bilateral absence of the vas deferens (CBAVD) (3, 4). Pre-testicular azoospermia arises when the hypothalamus and pituitary are not working properly to stimulate intrinsically normal testes in terms of both gonadal function (testosterone synthesis in the Leydig cells and spermatogenesis within the seminiferous tubules) (5). However, testicular azoospermia can also occur when the hypothalamus and pituitary are functioning normally, but there is a defect in the spermatogenic process resulting in no sperm production. Since the reproductive ductal structures are present and patent, the two mentioned etiologies fall under non-obstructive azoospermia (5).

The management of NOA or OA patients relies on conventional testicular sperm extraction (cTESE) or microscopic testicular sperm extraction (microTESE) followed by an intracytoplasmic sperm injection (ICSI) [artificial reproductive technology (ART)] (6). In NOA patients, sperm recovery is successful in only 50% of cases, and therefore the ability to predict the patients with a high probability of achieving a successful sperm retrieval would be of great value when counseling the patient and his partner (7, 8). However, several studies have tried to suggest predictors of a positive TESE (e.g. FSH concentration, Y chromosome microdeletions), but most concluded that testicular histology (diagnostic testicular biopsy) is best (9–16).

In this study, we investigated and compared cTESE, microTESE, and testicular histology outcomes in order to better understand the different criteria adopted by the two laboratory methodologies (IVF lab and pathological anatomy laboratory).

## 2 Materials and methods

### 2.1 Inclusion criteria

The present study is a retrospective analysis including patients who were referred to the IRCCS Humanitas Clinical Hospital, Fertility Center, between 2018 and 2020 for TESE treatment. We considered NOA, OA, azoospermia due to genetic factors (e.g. CBAVD, Klinefelter syndrome, or Y-chromosome microdeletions), mixed genesis azoospermia, idiopathic azoospermia, iatrogenic azoospermia, azoospermia due to cancer treatments, cryptozoospermia, necrozoospermia, altered sperm DNA fragmentation index (DFI), total sperm progressive motile count (TPMC) less than 1.000.000, retrograde ejaculation, anejaculation, and anorgasmia as inclusion criteria. The definitions of cryptozoospermia, necrozoospermia, anejaculation, and anorgasmia are described in the Laboratory Manual for the Examination and Processing of Human Semen by the World Health Organization (WHO) (2).

Regarding the evaluation of azoospermia, patients were confirmed to be azoospermic using at least two semen analyses. Their clinical history was recorded, including age, history of undescended testis, mumps orchitis, previous genito-urinary infection, radiotherapy, chemotherapy, surgical procedures, or exposure to gonadotoxins. The clinical examination included testicular size and consistency, epididymal dimension, and the presence of a varicocele. Follicle-stimulating hormone (FSH), luteinizing hormone (LH), and prolactin (PRL) concentrations were measured in all patients.

### 2.2 cTESE and microTESE (surgical approach)

All patients underwent surgical sperm retrieval under local or general anesthesia, based on whether it was a unilateral (one testicle opened) or bilateral (both testicles opened) surgical technique (same-day surgery). In both cTESE and microTESE, a median raphe incision was made in the scrotum, unless the testis was fixed in a position high in the scrotum. The tunica vaginalis was then carefully opened, allowing the epididymis and the testicle to be delivered.

In cTESE, a transverse 5-mm incision was made through the tunica albuginea with a blade scalpel, avoiding blood vessels. Then, the testis was gently squeezed in order to excise the seminiferous tubules which protrude with scissors (17) (limited to 10x6x6 mm for the unilateral procedures or 8x4x4 mm per part for the bilateral procedures).

MicroTESE is a costlier and more technically demanding procedure than cTESE, requiring an operating microscope and a skilled microsurgeon. It was reserved for NOA patients with very small testicular size (≤3 cm) to minimize testosterone therapy. After the testicle was delivered, the surgeon used the operating microscope, which allowed for the identification of the seminiferous tubules that are most likely to contain sperm (18); then, an incision was made in an avascular region. A wide transverse incision was made in an equatorial plane along the midportion with an ophthalmic knife to facilitate adequate exposure of the seminiferous tubules. These structures were examined to identify small loci of spermatogenesis (17).

The samples, derived from cTESE or microTESE, were immediately placed in sperm washing medium and an embryologist examined the tubules for the presence of spermatozoa.

During the same procedure, a histopathology specimen (3x3x3 mm) was placed in Bouin’s solution and sent for analysis. For adequate classification of spermatogenesis, the removed tissue should contain at least 100 seminiferous tubules (19, 20).

The tunica albuginea was closed with a continuous 5-0 monofilament nylon suture.

### 2.3 Sperm retrieval and cryopreservation

After opening the bioptic specimen and stressing the seminiferous tubules in a Petri dish (Falcon^®^) with Sperm Washing Medium (Fujifilm, Irvine Scientific), the suspension obtained was transferred to a centrifuge tube (Falcon^®^) and subjected to centrifugation at 475 RCF/10 minutes (Thermo Scientific Heraeus^®^ Megafuge^®^ 11R) in order to separate the liquid supernatant from the pellet. It was then resuspended in 0.5 mL Sperm Washing Medium (Fujifilm, Irvine Scientific) for the subsequent evaluation. We evaluated the samples’ spermatozoa concentration using a Makler^®^ counting chamber (Sefi Medical Instruments) and their viability using the eosin test. Regarding the sperm concentration, we placed 10µL of the sample in the center of the chamber and assessed the evaluation through a phase-contrast microscope (magnification 200x). The counting step was assessed twice per sample, both before and after cryopreservation. For all the samples in which the sperm concentration was too low to be assessed using the counting chamber, we counted spermatozoa (if present) per microscopic field on a slide (magnification 200x). Regarding viability, we gently mixed 5µL of the sample and 5µL of eosin solution on a slide, which was then covered with a coverslip. The evaluation was conducted using a phase-contrast microscope (magnification 400x), according to the WHO, 2010 (2). Where present, we evaluated the sperm motility as spermatozoa with twitching tail or wavy flagellar movement, as reported by Hosseini et al. (21). After the preliminary evaluations, we decided whether to cryopreserve the sample based on its suitability for assisted reproductive techniques (ART).

All the laboratory steps were executed by two embryologists: the first operator and the witness, whose role was to validate what the first operator observed.

### 2.4 Evaluation of patients based on their retrieval outcomes

The patients for whom we found a discordance between TESE and testicular histology outcomes were divided into two groups.

Group 1: patients for whom we found at least 1 sperm in the TESE retrieval, while the histology report did not find any spermatozoa or did not find enough compared to our evaluation.

Group 2: patients for whom we did not find any spermatozoa or did not find enough compared to what was detected in the testicular histology.

We investigated the pregnancy rate of patients who underwent ART belonging to Group 1.

Additionally, we examined only NOA patients, dividing them into two further subgroups: NOA subgroup 1, where we found at least 1 sperm in the TESE retrieval, and NOA subgroup 2, where we did not find any spermatozoa. We compared the hormonal profiles of these two subgroups using the Mann-Whitney test in order to check for any variance between the sample means as the data did not follow a Gaussian distribution.

### 2.5 Cytological evaluation of testicular spermatogenesis

Testicular biopsies were stained with Masson’s trichrome staining (22).

Based on the prevailing histopathological patterns, testicular histology was classified into normal spermatogenesis (10 or more spermatozoa per tubule), hypospermatogenesis (less than 10 spermatozoa per tubule), maturation arrest (the absence of the later stages of spermatogenesis), Sertoli cells only (SCO) (the absence of germ cells in the seminiferous tubules), and/or tubular sclerosis.

According to these patterns, we established a scoring system: 4) normal spermatogenesis; 3) hypospermatogenesis; 2) maturation arrest (complete); 1) SCO and/or Leydig cells only and/or tubular sclerosis.

## 3 Results

### 3.1 TESE patient baseline characteristics

In total, 526 patients who underwent TESE (309 unilateral and 217 bilateral procedures) at the Humanitas Fertility Center between 2018 and 2020 were recruited. Baseline characteristics (expressed as mean ± SD), indications for the TESE procedure, and ethnicities of the studied population are reported in **Table 1**. Not all the patients underwent ART with their partners nor did all the patients have a partner. Sperm viability was assessed for 331 of the 526 samples; 195 of the 526 samples had a sperm concentration that was too low, thus the viability could not be assessed.

**Table 1:** Baseline characteristics (expressed as mean ± SD), indication for TESE, and ethnicities of the studied population.

Baseline characteristics	Cases
TESE patients’ age (y)	38.9 ± 7.8 (n=526)
Female partners’ age (y)	34.6 ± 4.6 (n=432)
TESE patients’ BMI	25.8 ± 3.9 (n=526)
TESE samples’ viability (eosin test)	77.6 ± 8.1 (n=331)
Indications for TESE	Percentage (%)
NOA	118/526 (22,4%)
Cryptozoospermia	103/526 (19,6%)
OA	78/526 (14,8%)
Necrozoospermia	60/526 (11,4%)
Altered DFI	54/526 (10,3%)
TPMC < 1.000.000	35/526 (6,6%)
Genetic azoospermia	31/526 (5,9%)
Idiopathic azoospermia	15/526 (2,8%)
Anaejaculation	12/526 (2,3%)
Anorgasmia	7/526 (1,3%)
Azoospermia due to cancer treatments	5/526 (0,9%)
Iatrogenic azoospermia	3/526 (0,6%)
Mixed genesis azoospermia	3/526 (0,6%)
Retrograde ejaculation	2/526 (0,4%)
Ethnicity	Percentage (%)
Italian	471/526 (89,5%)
Romanian	10/526 (1,9%)
Albanian	10/526 (1,9%)
Moroccan	4/526 (0,8%)
Egyptian	3/526 (0,6%)
Moldavian	2/526 (0,4%)
English	2/526 (0,4%)
Peruvian	2/526 (0,4%)
Argentinian	2/526 (0,4%)
Serbian	2/526 (0,4%)
Syrian	2/526 (0,4%)
American	2/526 (0,4%)
Pakistani	2/526 (0,4%)
Ukrainian	1/526 (0,2%)
Polish	1/526 (0,2%)
Eritrean	1/526 (0,2%)
Philippine	1/526 (0,2%)
Chinese	1/526 (0,2%)
Belgian	1/526 (0,2%)
Dutch	1/526 (0,2%)
Ivorian	1/526 (0,2%)
Spanish	1/526 (0,2%)
Swiss	1/526 (0,2%)
Ecuadorian	1/526 (0,2%)
Indian	1/526 (0,2%)

### 3.2 Concordant and discordant outcomes between TESE and testicular histology

For 372 patients (372/526, 70.7%), concordance was found between the TESE and testicular histology outcomes; for 154 patients (154/526, 29.3%), discordance was found, as reported in **Supplementary Table S2** (**Supplementary Material**).

Regarding the disagreements, 146 cases (94.8%) were in Group 1 and 8 cases (5.2%) were in Group 2.

### 3.3 Pregnancy rate among Group 1 patients

The female partners of 61 of 146 patients (41.8%) with discordant outcomes between TESE and testicular histology (Group 1) who underwent ART achieved at least a pregnancy using a TESE cryopreserved sample.

### 3.4 NOA subgroups

The total number of NOA patients was 118, as reported in **Table 1**. As indicated in the materials and methods section, they were divided into two subgroups, based on their sperm retrieval.

In NOA subgroup 1 (80/118 patients, 67.8%), we found the following results, expressed as mean(± SD): age 35.1(± 5.6) years, follicle-stimulating hormone (FSH) 21.4(± 12.6) mIU/mL, luteinizing hormone (LH) 7.8(± 4.2) mIU/mL, testosterone (T) 5.3(± 4.4) ng/mL, prolactine (PRL) 11.6(± 11.2) ng/mL, right testicular volume 9.4±(3.7) mL, and left testicular volume 9.2±(3.6) mL. In this subgroup, the sperm retrieval outcomes of 24 patients agreed with the histopathological outcomes; the sperm retrieval outcomes of 56 patients disagreed with the histopathological outcomes.

In NOA subgroup 2 (38/118 patients, 32.2%), we found the following results, expressed as mean(± SD): age 38.1(± 6.4) years, FSH 22.3(± 9.1) mIU/mL, LH 8.4(± 4.9) mIU/mL, T 4.7(± 2.3) ng/mL, PRL 11.3(± 6.1) ng/mL, right testicular volume 10.2(± 4.0) mL, and left testicular volume 10.1(± 4.1) mL. The outcomes of all the patients in this subgroup agreed with the histopathological outcomes.

When comparing the hormonal profiles and testicular volumes in the NOA subgroups, we found no statistical difference as follows: FSH (*P*=0,2982), LH (*P*=0,5535), T (*P*=0,6231), PRL (*P*=0,4957), right testicular volume (*P*=0,2482), and left testicular volume (*P*=0,2138).

In NOA subgroup 1 (80/118 patients), we proceeded with cryopreservation for 65 patients. Subsequently, 49 patients underwent ART and the partners of 39 TESE patients (79.6%) became pregnant. The mean(± SD) age of the partners of the TESE patients was 32.2(± 4.2) years.

Considering all the NOA patients (118), we cryopreserved 65 TESE samples (55.1%).

## 4 Discussion

For many years testicular biopsy was considered the primary diagnostic tool for men with risk factors for testicular malignancy or male infertility in cases of unexplained infertility and azoospermia; however, currently, the guidelines for male infertility have limited the indications for a diagnostic testicular biopsy to the confirmation of obstructive or non-obstructive azoospermia (20). It may be part of the diagnostic process for infertile men, but it only confirms a disruption of spermatogenesis in cases where sperm count is low and follicle-stimulating hormone is high. Testicular biopsy is most useful in diagnosing obstructive azoospermia, where surgical repair can restore the presence of spermatozoa, increasing the chances of pregnancy. However, a standard testicular biopsy typically does not change the therapeutic options (20). Before the advent of the TESE technique, couples in which the male partner was diagnosed with azoospermia had to resort to heterologous fertilization through sperm donation (23–26). This is why it is important to emphasize the value of the combination of the TESE technique and an ICSI due to the possibility for NOA and OA patients to father biologically related children (8, 27, 28). Sperm retrieval rates in OA are approximately 100%, while in NOA they are approximately 60% (7, 8, 27, 28).

At our institute, TESE samples are meant to be cryopreserved. Indeed, no TESE procedures are performed at the time of oocyte pick-up. This is to prevent the partner’s stimulation from failing because it is possible that no sperm (destined for a subsequent ICSI) is found during the TESE, necessarily resulting in oocyte cryopreservation.

In this study, we compared the retrieval of 526 TESE samples with their histological outcomes from between 2018 and 2020. We found a discrepancy of almost 30% (29.3%, 154/526 samples) between the two techniques, as reported in **Supplementary Table S2** (**Supplementary Material**). In many cases, we cryopreserved testicular spermatozoa while the histological result reported the absence of germ cells in the seminiferous tubules (e.g., Sertoli cells only). Unfortunately, in 8 cases, we did not find any spermatozoa or did not find enough compared to the histology report, resulting in no sperm cryopreservation. This may be due to the fact that histological staining mainly highlights spermatozoa heads while embryologists need to observe the entire sperm structure to define it as one for the ICSI procedure. It is noteworthy that 61 female partners of TESE patients who were among those with discordant outcomes became pregnant with cryopreserved TESE samples.

As described in the materials and methods section, the histopathology specimen is smaller and above all different from that which is designated for TESE and our evidence highlights the presence of a heterogeneous histological area within the testis, representing a multifocal distribution of spermatogenesis (19, 29, 30). As other authors have reported, the histology of a small piece of tissue may not be representative of the tissue extracted or of the whole testis (31, 32). Furthermore, the tissue should not be squeezed since the testicular specimen may be disrupted, hampering a proper evaluation of the seminiferous tubules (20). Because of these reasons, regarding ART, some authors have concluded that testicular histology is of limited value (20, 33). The lack of clarity in the findings may stem from the incorrect identification of germ cells and confusing biopsy categorization, as the histological method is often inadequately described or omitted (20, 32). As Hessel M. et al. reported, to increase diagnostic accuracy, a larger biopsy should be sent to the histopathology laboratory, but the most representative and clinically relevant option may be to use a TESE cell suspension for a cytological evaluation of spermatogenesis (31).

Furthermore, we decided to specifically analyze the category of NOA patients, including retrieval rate, ART cycles, and pregnancy rate. We cryopreserved 65 of the 118 NOA TESE samples (55.1%) which was consistent with the literature (7). Moreover, our analysis revealed that while hormonal profiles and testicular volume may not predict TESE success, the overall pregnancy rate remains promising; indeed, 39/49 TESE patients’ partners who underwent ART became pregnant (79.6%). This suggests that TESE combined with an ICSI remains a viable and effective strategy for couples facing severe male infertility. The limitations of histological evaluation in reflecting the true spermatogenic potential reinforce the necessity of combining TESE with ART for an accurate diagnosis and treatment. Thus, in our analysis, we did not focus on the diagnoses of the female partners due to the main topic of the present retrospective study itself. Notably, a marked increase in impaired reproductive success is observed in women over 35 years of age (34). Furthermore, in our cohort, the age of the partners, expressed as mean(± SD), was 34.6(± 4.6) years. This is because when a man is diagnosed with severe spermatogenic failure (SPGF), such as NOA or OA, the immediate decision is to pursue ART with his partner; in particular, the latter does not wait to grow older. During the freezing and thawing processes, spermatozoa are exposed to physical and chemical stress, such as crystal formation or osmotic shock, which can affect membrane integrity or acrosome status (35, 36). Additional molecular alterations may accompany sperm cryopreservation (37). As a result, sperm cryopreservation leads to a decrease in sperm motility and sperm viability, increasing sperm DNA fragmentation (38, 39). However, Torra-Massana and colleagues found no difference between fresh and cryopreserved sperm in terms of oocyte fertilization, ongoing pregnancy, and live birth rates; moreover, comparable pregnancy rates can be achieved in OA and NOA cases (40, 41). It is important to emphasize that in the absence of sperm motility (in terms of twitching tail or wavy flagellar movement) in testicular sperm, hypo-osmotic swelling test (HOST), or laser-assisted immotile sperm selection (LAISS) are techniques recommended for before an ICSI to ensure a higher rate of success (21, 42). Finally, the pregnancy and delivery rates are strictly dependent on the age of the female partner (41).

However, a non-invasive diagnostic test that could predict sperm retrieval outcomes would greatly benefit the clinical management of NOA cases (7). In the past decade, significant advancements in high-throughput sequencing technologies have been made in biomedical research. Within this scenario, single-cell RNA sequencing (scRNA-seq) has emerged as a groundbreaking tool, uncovering the mechanisms behind testicular dysregulation and disorders (43, 44). A genetic cause has been identified in only 20% of infertile men with SPGF, leaving most cases unexplained and idiopathic (45). Growing evidence suggests that common genetic variants, such as single nucleotide polymorphisms (SNPs), may contribute to male infertility by disrupting the molecular processes that regulate spermatogenesis (46, 47). On this basis, researchers worldwide have moved to genome-wide association studies (GWASs) in the field of SPGF, identifying several risk variants for NOA susceptibility (47, 48).

From this perspective, germ cell maturation molecular patterns have been investigated in the literature. Germ cells undergo precise transitions between multiple cell types and cellular processes, including mitosis, meiosis, and spermiogenesis; the latter is accompanied by spermatid elongation and chromatin repackaging (49). The developmental process from spermatogonia into mature sperm depends on the expression of a range of genes in a precise temporal order (50). It was found that gonadotropin-regulated testicular RNA helicase (GRTH) is essential for the completion of spermatogenesis and male fertility. In particular, in the germ cells of male mice, there are two forms of GRTH: the non-phosphorylated and phosphorylated forms (pGRTH). GRTH knock-in (KI) mice with R242H mutation abolished pGRTH, leading to infertility (51). The same mutation was observed in 5.8% of Japanese men diagnosed with NOA (52).

In conclusion, to the best of our knowledge, our analysis confirmed that the TESE procedure combined with an ICSI remains the only strategy for couples facing severe male infertility. The choice of which testicular sperm to inject, especially when no wavy flagellar movement is observed in the sample, should be performed by an embryologist with vast expertise. However, a testicular biopsy is useful for diagnosing obstructive azoospermia where surgical repair can restore the presence of spermatozoa, increasing chances of pregnancy. Furthermore, novel molecular techniques and GWASs could help in understanding the matter of male infertility, which affects thousands of couples worldwide.

## Data Availability

The original contributions presented in the study are included in the article/**Supplementary Material**. Further inquiries can be directed to the corresponding author.
