# Supplementary material for: Rate of testicular histology failure in predicting successful testicular sperm extraction
Source: Front Endocrinol (Lausanne). 2024 Oct 10;15:1466675. doi: 10.3389/fendo.2024.1466675 (PMC11499163; doi:10.3389/fendo.2024.1466675)
Supplement: Supplementary file 1 [file Table1.docx]

| **HISTOLOGICAL SCORE:**  **1:** Sertoli Cells Only and/or Leydig cells only and/or tubular sclerosis  **2:** maturation arrest (complete)  **3:** hypospermatogenesis (less than 10 spermatozoa per tubule)  **4:** normal spermatogenesis (10 or more spermatozoa per tubule)  *: The asterisk-marked IDs indicate all the samples for whom we did not find any spermatozoa or found too few compared to what was detected in the testicular histology. | | | | | | |
| --- | --- | --- | --- | --- | --- | --- |
| **n.** | **ID** | **Diagnosis** | **TESE retrieval** | **Cryopreserved paillettes** | **Histological report** | **Histological score** |
| 1 | 2018_2 | NOA | DX: 100.000 spz/mL  SX: no sperm was detected | DX: 4  SX: 0 | DX: tubules consisting of Sertoli cells only  SX: tubules consisting of Sertoli cells only | DX: 1  SX: 1 |
| 2 | 2018_3 | Genetic Azoospermia | DX: 1 abnormal sperm every 30 microscopic fields of view was detected (200x)  SX: 1 abnormal sperm on the entire slide was detected | DX: 0  SX: 0 | DX: tubules consisting of Sertoli cells only or primary spermatocytes  SX: tubules consisting of Sertoli cells only | DX: 2  SX: 1 |
| 3 | 2018_13 | NOA | DX: 1 abnormal sperm on the entire slide was detected (200X)  SX: 1 sperm every 2 microscopic fields of view was detected (200x) | DX: 0  SX: 2 | DX: the majority of the tubules consisting of Sertoli cells and primary spermatocytes; spermatids were found in extremely rare tubules  SX: the majority of the tubules consisting of Sertoli cells and primary spermatocytes; spermatids were found in extremely rare tubules | DX: 2  SX: 2 |
| 4 | 2018_14 | Criptoazoospermia | DX: 400.000 spz/mL | DX: 4 | DX: tubules consisting of Sertoli cells only | DX: 1 |
| 5 | 2018_16* | High DFI | DX: 1 abnormal sperm every 40 microscopic fields of view was detected (200x)  SX: 1 abnormal sperm every 40 microscopic fields of view was detected (200x) | DX: 0  SX: 0 | DX: rare spermatozoa and some spermatids per tubule  SX: 5-7 spermatozoa and 7 spermatids per tubule | DX: 3  SX: 3 |
| 6 | 2018_25 | Idiopatic Azoospermia | DX: no sperm was detected  SX: 1 sperm every 10 microscopic fields of view was detected (200x) | DX: 0  SX: 1 | DX: almost all the tubules consisting of Sertoli cells, sometimes with some primary spermatocytes  SX: almost all the tubules consisting of Sertoli cells, sometimes with some primary spermatocytes and/or spermatids | DX: 2  SX: 2 |
| 7 | 2018_36 | Idiopatic Azoospermia | DX: 200.000 spz/mL  SX: 300.000 spz/mL | DX: 2  SX: 2 | DX: some spermatozoa and 10 spermatids per tubule  SX: the germ line maturation predominantly reaches the stage of primary spermatocyte/spermatid; extremely rare spermatozoa and some spermatids per tubule were found | DX: 3  SX: 3 |
| 8 | 2018_45 | Criptoazoospermia | DX: 300.000 spz/mL  SX: 100.000 spz/mL | DX: 2  SX: 1 | DX: germline maturation up to spermatid stage mostly, rare mature forms  SX: germline maturation up to spermatid stage mostly, rare mature forms | DX: 3  SX: 3 |
|  |  |  |  |  |  |  |
| **n.** | **ID** | **Diagnosis** | **TESE retrieval** | **Cryopreserved paillettes** | **Histological report** | **Histological score** |
| 9 | 2018_48 | Criptoazoospermia | DX: 1 abnormal sperm every 50 microscopic fields of view was detected (200x)  SX: 1 abnormal sperm every 40 microscopic fields of view was detected (200x) | DX: 0  SX: 0 | DX: almost all the tubules consisting of Sertoli cells and primary spermatocytes; extremely rare spermatids were found in extremely rare tubules  SX: almost all the tubules consisting of Sertoli cells and primary spermatocytes; some spermatids were found in extremely rare tubules | DX: 2  SX: 2 |
| 10 | 2018_51 | NOA | DX: 300.000 spz/mL  SX: no sperm was detected | DX: 3  SX: 0 | DX: complete maturation of the germ line, albeit reduced, rare spermatozoa and some spermatids  SX: tubules consisting of Sertoli cells only | DX: 3  SX: 1 |
| 11 | 2018_54* | NOA | DX: 1 sperm every 10 microscopic fields of view was detected (200x)  SX: 1 sperm every 10 microscopic fields of view was detected (200x) | DX: 1  SX: 1 | DX: rare spermatozoa and some spermatids per tubule  SX: some spermatozoa and spermatids per tubule | DX: 3  SX: 3 |
| 12 | 2018_62 | Criptoazoospermia | DX: 100.000 spz/mL  SX: 1 sperm every 2 microscopic fields of view was detected (200x) | DX: 1  SX: 1 | DX: rare marginal tubules in which a complete maturation of the germ line was observed  SX: in extremely rare tubules primary spermatocytes or spermatids were observed | DX: 3  SX: 2 |
| 13 | 2018_63 | TPMC<1.000.000 | DX: 100.000 spz/mL | DX: 2 | DX: maturational arrest with absence of mature spermatozoa | DX: 2 |
| 14 | 2018_64 | Idiopatic Azoospermia | DX: 1 abnormal sperm on the entire slide was detected  SX: 1 abnormal sperm on the entire slide was detected | DX: 0  SX: 0 | DX: tubules characterized by Sertoli cells and spermatocytes, rare spermatids  SX: tubules characterized by Sertoli cells and primary spermatocytes | DX: 2  SX: 2 |
| 15 | 2018_68 | Criptoazoospermia | DX: no sperm was detected  SX: 300.000 spz/mL | DX: 0  SX: 2 | DX: spermatocytes and extremely rare spermatids were observed in extremely rare tubules  SX: the germ line maturation reaches the spermatid stage, absence of mature forms | DX: 2  SX: 2 |
| 16 | 2018_77 | NOA | DX: 300.000 spz/mL  SX: 200.000 spz/mL | DX: 1  SX: 1 | DX: tubules consisting of Sertoli cells only  SX: some tubules characterized by Sertoli cells or primary spermatocytes | DX: 1  SX: 2 |
| 17 | 2018_79 | High DFI | DX: 1 sperm every 50 microscopic fields of view was detected (200x) | DX: 0 | DX: tubules characterized by Sertoli cells and primary spermatocytes; some spermatids were detected in rare tubules | DX: 2 |
| 18 | 2018_82 | NOA | DX: no sperm was detected  SX: 1 sperm on the entire slide was detected (200x) | DX: 0  SX: 0 | DX: tubules consisting of Sertoli cells only  SX: tubules consisting of Sertoli cells only | DX: 1  SX: 1 |
| 19 | 2018_86 | Genetic  azoospermia | DX: 1 sperm every 30 microscopic fields of view was detected (200x)  SX: 1 sperm every 30 microscopic fields of view was detected (200x) | DX: 1  SX: 1 | DX: tubular sclerosis and Sertoli cells were detected  SX: tubular sclerosis and Sertoli cells were detected | DX: 1  SX: 1 |
| 20 | 2018_89 | NOA | DX: 1 sperm every 50 microscopic fields of view was detected (200x) | DX: 0 | DX: tubules characterized by Sertoli cells and primary spermatocytes; some spermatids were detected in rare tubules | DX: 2 |
|  |  |  |  |  |  |  |
|  |  |  |  |  |  |  |
| **n.** | **ID** | **Diagnosis** | **TESE retrieval** | **Cryopreserved paillettes** | **Histological report** | **Histological score** |
| 21 | 2018_90 | NOA | DX: 100.000 spz/mL  SX: 1 sperm every 60 microscopic fields was detected (200x) | DX: 1  SX: 0 | DX: tubular sclerosis (50% of the tubules), germ line maturation predominantly up to spermatocyte stage (50% of the tubules), almost absent mature forms  SX: Sertoli cells, spermatocytes or spermatids | DX: 3  SX: 1 |
| 22 | 2018_94* | TPMC<1.000.000 | DX: 800.000 spz/mL | DX: 10 | DX: tubular sclerosis and Sertoli cells were observed | DX: 1 |
| 23 | 2018_95 | Criptoazoospermia | DX: 200.000 spz/mL  SX: 100.000 spz/mL | DX: 2  SX: 2 | DX: germ line maturation up to primary spermatocytes or spermatids, rare mature forms  SX: some spermatozoa and 12-15 spermatids per tubule | DX: 3  SX: 3 |
| 24 | 2018_97 | Genetic  Azoospermia | DX: 1 sperm every 20 microscopic fields of view was detected (200x)  SX: 1 sperm every 10 microscopic fields of view was detected (200x) | DX: 1  SX: 1 | DX: tubular sclerosis and Sertoli cells were observed  SX: tubular sclerosis, Sertoli cells and primary spermatocytes or spermatids were observed | DX: 1  SX: 2 |
| 25 | 2018_99 | NOA | DX: 1 sperm every 5 microscopic fields of view was detected (200x)  SX: 1 sperm every 5 microscopic fields of view was detected (200x) | DX: 1  SX: 1 | DX: most of the tubules characterized by Sertoli cells; primary spermatocytes were observed in some tubules  SX: most of the tubules characterized by Sertoli cells; primary spermatocytes were observed in some tubules | DX: 2  SX: 2 |
| 26 | 2018_101 | Criptoazoospermia | SX: 1 sperm every 20 microscopic fields of view was detected (200x | SX: 1 | SX: some spermatozoa and 15-18 spermatids per tubule | SX: 3 |
| 27 | 2018_104 | NOA | DX: 1 sperm every 15 microscopic fields of view was detected (200x)  SX: 1 sperm on the entire slide was detected | DX: 1  SX: 0 | DX: primary spermatocytes and spermatids were observed in extremely rare tubules  SX: tubules consisting of Sertoli cells only | DX: 2  SX: 1 |
| 28 | 2018_106 | Genetic  Azoospermia | DX: 1 sperm every 10 microscopic fields of view was detected (200x) | DX: 2 | DX: tubules consisting of Sertoli cells only | DX: 1 |
| 29 | 2018_109 | Criptoazoospermia | DX: 1 sperm every 50 microscopic fields of view was detected (200x) | DX: 0 | DX: primary spermatocytes and spermatids were observed in extremely rare tubules | DX: 2 |
| 30 | 2018_117 | NOA | DX: 1 sperm every 2 microscopic fields of view was detected (200x)  SX: 200.000 | DX: 1  SX: 1 | DX: primary spermatocytes or spermatids were observed in extremely rare tubules  SX: Sertoli cells (50% of the tubules) and primary spermatocytes or spermatids (50% of the tubules) | DX: 2  SX: 2 |
| 31 | 2018_121 | Criptoazoospermia | DX: 1 sperm every 8 microscopic fields of view was detected (200x)  SX: 200.000 spz/mL | DX: 1  SX: 2 | DX: germ line maturation up to spermatid  SX: uneven germ line maturation up to spermatid stage and focally to spermtozoa | DX: 2  SX: 3 |
| 32 | 2018_123 | NOA | DX: 1 sperm every 10 microscopic fields of view was detected (200x)  SX: 1 sperm every microscopic field of view was detected (200x) | DX: 2  SX: 1 | DX: Sertoli cells; in some tubules, primary spermatocytes or spermatids were observed  SX: tubules consisting of Sertoli cells only | DX: 2  SX: 1 |
| 33 | 2018_124 | NOA | DX: 1 sperm every 50 microscopic fields of view was detected (200x)  SX: 1 sperm every 50 microscopic fields of view was detected (200x) | DX: 0  SX: 0 | DX: primary spermatocytes were observed in four tubules  SX: primary spermatocytes and spermatids were observed in less than 5% of the tubules | DX: 2  SX: 2 |
| **n.** | **ID** | **Diagnosis** | **TESE retrieval** | **Cryopreserved paillettes** | **Histological report** | **Histological score** |
| 34 | 2018_128 | NOA | DX: 200.000 spz/mL  SX: 1 sperm every 50 microscopic fields of view was detected (200x) | DX: 1  SX: 0 | DX: in extremely rare tubules, primary spermatocytes were observed, and in even rarer tubules, some mature forms of the germ line were observed  SX: primary spermatocytes were observed in extremely rare tubules | DX: 3  SX: 2 |
| 35 | 2018_132 | DFI | DX: 400.000 spz/mL  SX: 1 sperm on the entire slide was detected | DX: 4  SX: 0 | DX: rare spermatozoa and spermatids per tubule  SX: Sertoli cells, primary spermatocytes and rare spermatids | DX: 3  SX: 2 |
| 36 | 2018_137 | Idiopatic Azoospermia | DX: 400.000 spz/mL  SX: 400.000 spz/mL | DX: 2  SX: 2 | DX: germ line maturation up to primary spermatocytes or spermatids  SX: rare spermatozoa and spermatids per tubule | DX: 2  SX: 3 |
| 37 | 2018_140 | Genetic  Azoospermia | DX: no sperm was detected  SX: 1 sperm every 30 microscopic fields of view was detected (200x) | DX: 0  SX: 1 | DX: tubules consisting of Sertoli cells only  SX: tubules consisting of Sertoli cells only | DX: 1  SX: 1 |
| 38 | 2018_142 | OA | SX: 1 sperm every 30 microscopic fields of view was detected (200x) | SX: 1 | SX: tubular sclerosis or Seroli cells were detected | SX: 1 |
| 39 | 2018_145 | Genetic  Azoospermia | DX: 1 sperm on the entire slide was detected  SX: no sperm was detected | DX: 1  SX: 1 | DX: tubules consisting of Sertoli cells only  SX: tubules consisting of Sertoli cells only | DX: 1  SX: 1 |
| 40 | 2018_148 | NOA | SX: 1 sperm every 20 microscopic fields of view was detected (200x) | SX: 1 | SX: tubules consisting of Sertoli cells only | SX: 1 |
| 41 | 2018_150 | Genetic  Azoospermia | DX: 200.000 spz/mL  SX: 300.000 spz/mL | DX: 1  SX: 1 | DX: germ line maturation up to spermatid stage  SX: germ line maturation up to spermatid stage | DX: 2  SX: 2 |
| 42 | 2018_152 | NOA | DX: 400.000 spz/mL  SX: 100.000 spz/mL | DX: 3  SX: 1 | DX: extremely rare spermatozoa and spermatids per tubule  SX: extremely rare spermatozoa and spermatids per tubule | DX: 3  SX: 3 |
| 43 | 2018_158 | NOA | DX: 1 sperm every 25 microscopic fields of view was detected (200x)  SX: 1 sperm every 5 microscopic fields of view was detected (200x) | DX: 0  SX: 1 | DX: tubules consisting of Sertoli cells only  SX: germ line maturation up to spermatid stage in extremely rare tubules | DX: 1  SX: 2 |
| 44 | 2018_169 | Criptoazoospermia | DX: 1 sperm every 30 microscopic fields of view was detected (200x)  SX: 1 sperm on the entire slide was detected | DX: 0  SX: 0 | DX: Sertoli cells, primary spermatocytes and extremely rare spermatids were detected  SX: Sertoli cells, primary spermatocytes and extremely rare spermatids were detected | DX: 2  SX: 2 |
| 45 | 2018_173 | Necrozoospermia | DX: 400.000 spz/mL  SX: 500.000 spz/mL | DX: 6  SX: 4 | DX: germ line maturation up to primary spermatocytes or spermatids, extremely rare mature forms were detected  SX: germ line maturation up to primary spermatocytes or spermatids, extremely rare mature forms were detected | DX: 3  SX: 3 |
| 46 | 2018_175 | NOA | DX: 200.000 spz/mL | DX: 2 | DX: tubules consisting of Sertoli cells only | DX: 1 |
| 47 | 2018_197 | Criptoazoospermia | DX: no sperm was detected  SX: 100.000 spz/mL | DX: 0  SX: 1 | DX: Sertoli cells and primary spermatocytes were detected  SX: Sertoli cells and primary spermatocytes were detected | DX: 2  SX: 2 |
| 48 | 2018_198 | Necrozoospermia | DX: 300.000 spz/mL | DX: 4 | DX: Sertoli cells and primary spermatocytes, rare spermatids were detected in rare tubules | DX: 2 |
| **n.** | **ID** | **Diagnosis** | **TESE retrieval** | **Cryopreserved paillettes** | **Histological report** | **Histological score** |
| 49 | 2018_199 | NOA | SX: 100.000 spz/mL | SX: 1 | SX: some primary spermatocytes were detected in extremely rare tubules | DX: 2 |
| 50 | 2019_18 | Criptoazoospermia | DX: 1 abnormal sperm every 10 microscopic fields of view was detected (200x)  SX: 100.000 spz/mL | DX: 0  SX: 1 | DX: Sertoli cells and primary spermatocytes, rare spermatids were detected in some tubules  SX: some spermatids and rare spermatozoa | DX: 2  SX: 3 |
| 51 | 2019_19 | Criptoazoospermia | SX: 100.000 spz/mL | SX: 2 | SX: rare primary spermatocytes and spermatids per tubule | SX: 2 |
| 52 | 2019_20 | Necrozoospermia | DX: 400.000 spz/mL  SX: 300.000 spz/mL | DX: 3  SX: 2 | DX: 5-7 spermatozoa and 10-12 spermatids per tubule  SX: germ line maturation up to primary spermatocyte or spermatid stage mostly, rare mature forms | DX: 3  SX: 3 |
| 53 | 2019_32 | NOA | DX: no sperm was detected  SX: 1 sperm every 25 microscopic fields of view was detected (200x) | DX: 0  SX: 1 | DX: tubules consisting of Sertoli cells only  SX: tubules consisting of Sertoli cells only | DX: 1  SX: 1 |
| 54 | 2019_33 | TPMC<1.000.000 | DX: 1 sperm on the entire slide was detected  SX: 1 sperm every 20 microscopic fields of view was detected (200x) | DX: 0  SX: 0 | DX: some spermatozoa and 7-10 spermatids per tubule  SX: 7-10 spermatozoa and 7-10 spermatids per tubule | DX: 3  SX: 3 |
| 55 | 2019_38* | Criptoazoospermia | DX: 1 abnormal sperm every 20 microscopic fields of view was detected (200x) | DX: 0 | DX: 10-15 spermatozoa and 10-15 spermatids per tubule | DX: 4 |
| 56 | 2019_40 | Criptoazoospermia | DX: 400.000 spz/mL  SX: 1 sperm every 5 microscopic fields of view was detected (200x) | DX: 1  SX: 1 | DX: some spermatozoa and spermatids per tubule  SX: tubular sclerosis and Sertoli cells were detected | DX: 3  SX: 1 |
| 57 | 2019_49 | Idiopatic Azoospermia | DX: no sperm was detected  SX: no sperm was detected | DX: 0  SX: 0 | DX: germ line maturation up to primary spermatocyte or spermatid stage, almost absent mature forms  SX: germ line maturation up to spermatid stage, almost absent mature forms | DX: 3  SX: 3 |
| 58 | 2019_50 | NOA | DX: 2 spermatozoa on the entire slide were detected  SX: 1 sperm every 5 microscopic fields of view was detected (200x) | DX: 0  SX: 1 | DX: germ line maturation up to primary spermatocyte or spermatid stage, extremely rare mature forms  SX: tubular sclerosis | DX: 3  SX: 1 |
| 59 | 2019_52 | NOA | DX: 200.000 spz/mL  SX: 200.000 spz/mL | DX: 1  SX: 2 | DX: tubules consisting of Sertoli cells only  SX: complete germ line maturation in 5-10% of the tubules | DX: 1  SX: 3 |
| 60 | 2019_55 | NOA | DX: 1 sperm every 30 microscopic fields of view was detected (200x)  SX: 300.000 spz/mL | DX: 0  SX: 2 | DX: tubules constituted of Seroli cells; primary spermatocytes and spermatids were detected in one tubule  SX: complete germ line maturation in 5-10% of the tubules | DX: 2  SX: 3 |
| 61 | 2019_57 | Mixed Genesis Azoospermia | DX: 400.000 spz/mL | DX: 2 | DX: germ line maturation up to primary spermatocyte or spermatid stage, almost absent mature forms | DX: 3 |
| 62 | 2019_58 | NOA | DX: 400.000 spz/mL  SX: 2 spermatozoa on the entire slide were detected | DX: 2  SX: 0 | DX: germ line maturation up to primary spermatocyte or spermatid stage, rare mature forms in rare tubules  SX: germ line maturation up to primary spermatocyte or spermatid stage, rare mature forms in rare tubules | DX: 3  SX: 3 |
| **n.** | **ID** | **Diagnosis** | **TESE retrieval** | **Cryopreserved paillettes** | **Histological report** | **Histological score** |
| 63 | 2019_63 | TPMC<1.000.000 | DX: 500.000 spz/mL | DX: 4 | DX: germ line maturation up to primary spermatocyte or spermatid stage, rare spermatozoa in rare tubules | DX: 3 |
| 64 | 2019_69 | NOA | DX: 1 sperm every 30 microscopic fields of view was detected (200x)  SX: 1 sperm every 50 microscopic fields of view was detected (200x) | DX: 0  SX: 0 | DX: tubules consisting of Sertoli cells only  SX: tubules consisting of Sertoli cells only | DX: 1  SX: 1 |
| 65 | 2019_70 | NOA | DX: 300.000 spz/mL  SX: 1 sperm on the entire slide was detected | DX: 4  SX: 0 | DX: 75% of the tubules consisting of Sertoli cells, complete germ line maturation observed in 25% of the tubules  SX: tubules consisting of Sertoli cells only | DX: 3  SX: 1 |
| 66 | 2019_73 | NOA | DX: 1 sperm on the entire slide was detected  SX: 4 spermatozoa on the entire slide were detected | DX: 0  SX: 0 | DX: tubular sclerosis and Sertoli cells detected in rare tubules  SX: tubular sclerosis and Sertoli cells detected in rare tubules | DX: 1  SX: 1 |
| 67 | 2019_74 | TPMC<1.000.000 | DX: 1 abnormal sperm every 15 microscopic fields of view was detected (200x) | DX: 0 | DX: tubules consisting of Sertoli cells and primary spermatocytes predominantly, rare spermatids in some tubules | DX: 2 |
| 68 | 2019_77 | OA | DX: 1 sperm on the entire slide was detected  SX: 100.000 spz/mL | DX: 0  SX: 1 | DX: tubules consisting of Sertoli cells and primary spermatocytes  SX: tubules consisting of Sertoli cells and primary spermatocytes, rare spermatids in some tubules | DX: 2  SX: 2 |
| 69 | 2019_81 | Criptoazoospermia | DX: 1 sperm every 10 microscopic fields of view was detected (200x)  SX: 400.000 spz/mL | DX: 1  SX: 2 | DX: 90% of the tubules consisting of Sertoli cells only, 10% of the tubules consisting of complete germ line maturation  SX: primary spermatocytes and spermatids detected in extremely rare tubules | DX: 3  SX: 2 |
| 70 | 2019_92* | Criptoazoospermia | DX: 1 sperm per microscopic field of view was detected (200x)  SX: no sperm was detected | DX: 2  SX: 0 | DX: some spermatozoa and 15 spermatids per tubule  SX: some spermatozoa and spermatids per tubule | DX: 3  SX: 3 |
| 71 | 2019_95 | Criptoazoospermia | DX: 1 abnormal sperm every 15 microscopic fields of view was detected (200x)  SX: 1 sperm on the entire slide was detected | DX: 0  SX: 0 | DX: 95% of the tubules consisting of Sertoli cells only, 5% of the tubules consisting of complete germ line maturation  SX: tubular sclerosis and Sertoli cells | DX: 3  SX: 1 |
| 72 | 2019_99 | Criptoazoospermia | DX: 1 sperm every 2 microscopic fields was detected (200x) | DX: 1 | DX: reduced germ line maturation up to primary spermatocyte and spermatid in some tubules | DX: 2 |
| 73 | 2019_100 | NOA | DX: 200.000 spz/mL  SX: 500.000 spz/mL | DX: 1  SX: 2 | DX: tubules consisting of Sertoli cells only  SX: some spermatozoa and spermatids per tubule | DX: 1  SX: 3 |
| 74 | 2019_105 | Criptoazoospermia | DX: 1 abnormal sperm every 20 microscopic fields was detected (200x)  SX: 400.000 spz/mL | DX: 0  SX: 2 | DX: tubules consisting of Sertoli cells only and primary spermatocytes  SX: germ line maturation up to primary spermatocyte or spermatid stage, extremely rare mature forms | DX: 2  SX: 3 |
| 75 | 2019_107 | Azoospermia due to cancer treatments | SX: 1 abnormal sperm every 50 microscopic fields was detected (200x) | SX: 0 | SX: tubules consisting of Sertoli cells only | SX: 1 |
|  |  |  |  |  |  |  |
| **n.** | **ID** | **Diagnosis** | **TESE retrieval** | **Cryopreserved paillettes** | **Histological report** | **Histological score** |
| 76 | 2019_118 | Criptoazoospermia | DX: 400.000 spz/mL  SX: 300.000 spz/mL | DX: 2  SX: 2 | DX: extremely rare tubules consisting of extremely rare spermatozoa  SX: some spermatozoa and spermatids per tubule | DX: 3  SX: 3 |
| 77 | 2019_120 | High DFI | DX: 100.000 spz/mL  SX: 400.000 spz/mL | DX: 2  SX: 3 | DX: 50% of the tubules consisting of Sertoli cells only and 50% consisting of germ line maturation up to primary spermatocyte or spermatid stage  SX: extremely rare tubules consisting of complete germ line maturation | DX: 2  SX: 3 |
| 78 | 2019_121 | Genetic Azoospermia | DX: 1 sperm every 20 microscopic fields was detected (200x)  SX: 1 sperm every 10 microscopic fields was detected (200x) | DX: 1  SX: 1 | DX: tubules consisting of Sertoli cells only  SX: only one tubule showed a complete germline maturation | DX: 1  SX: 3 |
| 79 | 2019_125 | DFI | DX: 400.000 spz/mL | DX: 4 | DX: germ line maturation up to spermatid stage, extremely rare mature forms | DX: 3 |
| 80 | 2019_128 | NOA | DX: 100.000 spz/mL  SX: 100.000 spz/mL | DX: 1  SX: 1 | DX: tubular sclerosis or Sertoli cells only  SX: tubular sclerosis or Sertoli cells only | DX: 1  SX: 1 |
| 81 | 2019_132 | Anejaculation | DX: 500.000 spz/mL | DX: 4 | DX: rare spermatozoa and some spermatids per tubule | DX: 3 |
| 82 | 2019_134 | NOA | DX: 1 sperm every 40 microscopic fields was detected (200x) | DX: 0 | DX: 1% of the tubules showed spermatids | DX: 2 |
| 83 | 2019_136 | NOA | DX: 100.000 spz/mL  SX: 100.000 spz/mL | DX: 1  SX: 1 | DX: germline maturation up to primary spermatocyte, spermatids in some tubules  SX: germline maturation up to primary spermatocyte or spermatide stage, some mature forms in some tubules | DX: 2  SX: 3 |
| 84 | 2019_141* | Criptoazoospermia | DX: 1 sperm every 8 microscopic fields was detected (200x)  SX: 1 sperm every 10 microscopic fields was detected (200x) | DX: 1  SX: 1 | DX: some spermatozoa and spermatids per tubule  SX: rare spermatozoa and some spermatids per tubule | DX: 3  SX: 3 |
| 85 | 2019_157 | NOA | DX: 1 abnormal sperm every 50 microscopic fields was detected (200x)  SX: 300.000 spz/mL | DX: 0  SX: 3 | DX: tubules consisting of Sertoli cells only  SX: some spermatozoa and spermatids per tubule | DX: 1  SX: 3 |
| 86 | 2019_158 | NOA | SX: 1 abnormal sperm every 50 microscopic fields was detected (200x) | SX: 0 | SX: tubules consisting of Sertoli cells only | SX: 1 |
| 87 | 2019_166 | NOA | DX: 100.000 spz/mL  SX: 300.000 spz/mL | DX: 1  SX: 3 | DX: 85%-90% of the tubules consisting of Sertoli cells only, 15%-10% of the tubules showing complete germline maturation  SX: extremely rare tubules consisting of complete germline maturation | DX: 3  SX: 3 |
| 88 | 2019_175 | OA | DX: 500.000 spz/mL | DX: 8 | DX: rare spermatozoa and 10 spermatids per tubule | DX: 3 |
| 89 | 2019_176 | Criptoazoospermia | SX: 400.000 spz/mL | SX: 3 | SX: rare spermatozoa and some spermatids per tubule | SX: 3 |
| 90 | 2019_181 | Criptoazoospermia | DX: 400.000 spz/mL | DX: 2 | DX: germline maturation up to primary spermatocyte predominantly, rare spermatids and mature forms | DX: 3 |
| 91 | 2019_188* | Idiopatic Azoospermia | DX: no sperm was found  SX: no sperm was found | DX: 0  SX: 0 | DX: rare spermatozoa and some spermatids per tubule  SX: germline maturation up to spermatid stage, almost absent the mature forms | DX: 3  SX: 3 |
|  |  |  |  |  |  |  |
| **n.** | **ID** | **Diagnosis** | **TESE retrieval** | **Cryopreserved paillettes** | **Histological report** | **Histological score** |
| 92 | 2019_190 | NOA | DX: 1 abnormal sperm on the entire slide was detected (200x)  SX: 1 abnormal sperm every 40 microscopic fields was detected (200x) | DX: 0  SX: 0 | DX: 50% of the tubules consisting of complete germline maturation, 35% consisting of Sertoli cells only or primary spermatocytes, 15% consisting of tubular sclerosis  SX: germline maturation up to primary spermatocyte stage, extremely rare spermatids | DX: 3  SX: 2 |
| 93 | 2020_2 | Criptoazoospermia | DX: 300.000 spz/mL  SX: 1 abnormal sperm on the entire slide was found (200x) | DX: 3  SX: 0 | DX: sections of didymus characterized by the majority of tubules in sclerosis, some of which are sites of intraluminal proliferation of atypical elements consistent with in situ germ cell neoplasia (IGCN). Some tubules with complete maturation of the germinal line are localized marginally to the specimen.  SX: sections of didymus characterized by the majority of tubules in sclerosis, some of which are sites of intraluminal proliferation of atypical elements consistent with in situ germ cell neoplasia (IGCN). Not clear infiltrative aspects. | DX: 3  SX: 1 |
| 94 | 2020_6 | NOA | DX: 700.000 spz/mL  SX: 800.000 spz/mL | DX: 3  SX: 3 | DX: germline maturation up to spermatid stage, extremely rare the mature forms  SX: germline maturation up to spermatid stage, extremely rare the mature forms | DX: 3  SX: 3 |
| 95 | 2020_9 | NOA | DX: 200.000 spz/mL  SX: 400.000 spz/mL | DX: 2  SX: 2 | DX: complete germline maturation in extremely rare tubules  SX: germline maturation up to spermatid stage predominantly, rare mature forms | DX: 3  SX: 3 |
| 96 | 2020_12 | DFI | DX: 200.000spz/mL | DX: 3 | DX: germline maturation up to spermatid stage predominantly, almost absent the mature forms | DX: 3 |
| 97 | 2020_13 | NOA | DX: 1 sperm every 20 microscopic fields was detected (200x)  SX: 1 sperm every 20 microscopic fields was detected (200x) | DX: 1  SX: 1 | DX: majority of the tubules consisting of Sertoli cells only, extremely rare primary spermatocytes and spermatids  SX: majority of the tubules consisting of Sertoli cells only, extremely rare primary spermatocytes and spermatids | DX: 2  SX: 2 |
| 98 | 2020_15 | Necrozoospermia | SX: 500.000 spz/mL | SX: 7 | SX: rare spermatozoa and 10 spermatids per tubule | DX: 3 |
| 99 | 2020_16 | Criptoazoospermia | DX: 1 abnormal sperm every 20 microscopic fields (200x)  SX: 100.000 spz/mL | DX: 0  SX: 1 | DX: some spermatozoa and spermatids per tubule  SX: germline maturation up to spermatid stage predominantly, some tubules consisting of Sertoli cells only and primary spermatocytes | DX: 3  SX: 2 |
| 100 | 2020_17 | TPMC<1.000.000 | DX: 1 sperm every 3 microscopic fields (200x)  SX: 400.000 spz/mL | DX: 1  SX: 2 | DX: germline maturation up to spermatid stage, almost absent the mature forms  SX: germline maturation up to spermatid stage, rare mature forms | DX: 3  SX: 3 |
| 101 | 2020_18 | Genetic Azoospermia | DX: no sperm was detected  SX: 1 abnormal sperm on the entire slide was detected (200x) | DX: 0  SX: 0 | DX: tubules consisting of Sertoli cells only  SX: tubules consisting of Sertoli cells only | DX: 1  SX: 1 |
| 102 | 2020_20 | Genetic Azoospermia | DX: no sperm was detected  SX: 1 abnormal sperm on the entire slide was detected (200x) | DX: 0  SX: 0 | DX: tubules consisting of Sertoli cells only  SX: tubules consisting of Sertoli cells only | DX: 1  SX: 1 |
| 103 | 2020_30* | NOA | DX: 1 abnormal sperm on the entire slide was detected (200x)  SX: no sperm was detected | DX: 0  SX: 0 | DX: rare tubules consisting of complete germline maturation  SX: rare tubules consisting of complete germline maturation | DX: 3  SX: 3 |
| **n.** | **ID** | **Diagnosis** | **TESE retrieval** | **Cryopreserved paillettes** | **Histological report** | **Histological score** |
| 104 | 2020_34 | NOA | DX: 1 sperm every 20 microscopic fields was detected (200x)  SX: 1 sperm every 10 microscopic fields was detected (200x) | DX: 1  SX: 1 | DX: sections of didymus carachterized by tubular sclerosis, some tubules consisting of Sertoli cells only and primary spermatocytes  SX: majority of the tubules consisting of Sertoli cells only and primary spermatocytes, extremely rare tubules consisting of complete germline maturation | DX: 2  SX: 3 |
| 105 | 2020_37 | Criptoazoospermia | SX: 300.000 spz/mL | SX: 3 | SX: rare spermatozoa and some spermatids per tubule | SX: 3 |
| 106 | 2020_39 | NOA | DX: no sperm was detected  SX: 1 abnormal sperm on the entire slide was detected (200x) | DX: 0  SX: 0 | DX: tubules consisting of Sertoli cells only  SX: tubules consisting of Sertoli cells only | DX: 1  SX: 1 |
| 107 | 2020_42 | Genetic Azoospermia | DX: 1 abnormal sperm every 40 microscopic fields was detected (200x) | DX: 0 | DX: majority of the tubules consisting of Sertoli cells only, some tubules consisting of germline maturation up to spermatid stage | DX: 2 |
| 108 | 2020_43 | NOA | DX: 1 abnormal sperm on the entire slide was detected (200x)  SX: 1 sperm every 5 microscopic fields was detected (200x) | DX: 0  SX: 1 | DX: tubules consisting of Sertoli cells only  SX: tubules consisting of Sertoli cells only, extremely rare primary spermatocytes | DX: 1  SX: 2 |
| 109 | 2020_47 | Criptoazoospermia | DX: 1 abnormal sperm every 30 microscopic fields (200x)  SX: no sperm was detected | DX: 0  SX: 0 | DX: tubules consisting of Sertoli cells only  SX: tubules consisting of Sertoli cells only | DX: 1  SX: 1 |
| 110 | 2020_48 | NOA | DX: no sperm was detected  SX: 1 abnormal sperm on the entire slide was detected (200x) | DX: 0  SX: 0 | DX: tubules consisting of Sertoli cells only  SX: tubules consisting of Sertoli cells only | DX: 1  SX: 1 |
| 111 | 2020_49 | NOA | DX: 1 abnormal sperm every 50 microscopic fields (200x)  SX: 1 abnormal sperm every 50 microscopic fields (200x) | DX: 0  SX: 0 | DX: tubules consisting of Sertoli cells only, extremely rare primary spermatocytes and spermatids  SX: germline maturation up to primary spermatocyte or spermatid stage | DX: 2  SX: 2 |
| 112 | 2020_50 | NOA | DX: 1 sperm every 5 microscopic fields (200x)  SX: 1 abnormal sperm every 50 microscopic fields (200x) | DX: 1  SX: 0 | DX: 70% of the tubules consisting of Sertoli cells only, 30% consisting of complete germline maturation  SX: tubules consisting of Sertoli cells only, extremely rare tubules consisting of germline maturation up to spermatid stage | DX: 3  SX: 2 |
| 113 | 2020_51 | Criptoazoospermia | DX: 1 abnormal sperm on the entire slide was detected (200x)  SX: 1 sperm every 5 microscopic fields (200x) | DX: 0  SX: 1 | DX: tubules consisting of Sertoli cells only  SX: tubules consisting of Sertoli cells only | DX: 1  SX: 1 |
| 114 | 2020_56 | NOA | DX: 1 abnormal sperm every 10 microscopic fields was detected (200x)  SX: 1 sperm every 5 microscopic fields was detected (200x) | DX: 0  SX: 1 | DX: germline maturation up to primary spermatocyte stage predominantly, rare spermatids in some tubules  SX: germline maturation up to primary spermatocyte stage predominantly, rare spermatids in some tubules | DX: 2  SX: 2 |
|  |  |  |  |  |  |  |
| **n.** | **ID** | **Diagnosis** | **TESE retrieval** | **Cryopreserved paillettes** | **Histological report** | **Histological score** |
| 115 | 2020_60 | NOA | DX: 1 sperm every 3 microscopic fields was detected (200x)  SX: no sperm was detected | DX: 1  SX: 0 | DX: rare tubules consisting of germline maturation up to primary spermatocyte or spermatid stage  SX: tubules consisting of Sertoli cells only | DX: 2  SX: 1 |
| 116 | 2020_61 | NOA | DX: 1 sperm every 15 microscopic fields was detected (200x) | DX: 1 | DX: majority of the tubules consisting of Sertoli cells only, extremely rare tubules consisting germline maturation up to primary spermatocyte or spermatid stage | DX: 2 |
| 117 | 2020_62 | NOA | DX: 1 sperm every 5 microscopic fields was detected (200x)  SX: 1 sperm every 10 microscopic fields was detected (200x) | DX: 1  SX: 1 | DX: 60% of the tubules consisting of tubular sclerosis or Sertoli cells only, 40% of the tubules consisting of extremely rare mature forms  SX: tubular sclerosis or tubules consisting of Sertoli cells only, extremely rare primary spermatocytes and spermatids | DX: 3  SX: 2 |
| 118 | 2020_63 | OA | DX: 700.000 spz/mL | DX: 8 | DX: extremely rare spermatozoa and some spermatids per tubule | DX: 3 |
| 119 | 2020_65 | Criptoazoospermia | DX: 1 abnormal sperm every 100 microscopic fields was detected (200x)  SX: 1 sperm every 20 microscopic fields was detected (200x) | DX: 0  SX: 1 | DX: tubules consisting of Sertoli cells only  SX: tubules consisting of Sertoli cells only | DX: 1  SX: 1 |
| 120 | 2020_69 | TPMC<1.000.000 | DX: 400.000 spz/mL | DX: 3 | DX: tubules consisting of germline maturation up to spermatid stage predominantly, almost absent the mature forms | DX: 2  SX: 2 |
| 121 | 2020_71 | OA | DX: 1.000.000 spz/mL | DX: 8 | DX: tubules consisting of rare spermatozoa and 10-12 spermatids per tubules | DX: 3 |
| 122 | 2020_74 | Criptoazoospermia | SX: 1 sperm every 5 microscopic fields was detected (200x) | SX: 1 | SX: tubules consisting of Sertoli cells only, primary spermatocytes in some tubules | SX: 2 |
| 123 | 2020_75 | TPMC<1.000.000 | SX: 1 abnormal sperm every 5 microscopic fields was detected (200x) | SX: 0 | SX: tubules consisting of Sertoli cells only, rare primary spermatocytes | SX: 2 |
| 124 | 2020_78 | Criptoazoospermia | DX: 400.000 spz/mL  SX: 500.000 spz/mL | DX: 4  SX: 4 | DX: germline maturation up to spermatid stage predominantly, rare spermatozoa  SX: germline maturation up to primary spermatocyte or spermatid stage predominantly, rare spermatozoa | DX: 3  SX: 3 |
| 125 | 2020_79 | Idiopatic  Azoospermia | DX: 1 abnormal sperm on the entire slide was detected (200x)  SX: 1 abnormal sperm on the entire slide was detected (200x) | DX: 0  SX: 0 | DX: tubules consisting of germline maturation up to primary spermatocyte stage predominantly, rare spermatids  SX: tubules consisting of germline maturation up to primary spermatocyte stage predominantly, rare spermatids | DX: 2  SX: 2 |
| 126 | 2020_81 | Necrozoospermia | DX: 500.000 spz/mL | DX: 5 | DX: germline maturation up to spermatid stage, rare spermatozoa in some tubules | DX: 3 |
| 127 | 2020_82 | Necrozoospermia | DX: 300.000 spz/mL | DX: 2 | DX: germline maturation up to spermatid stage predominantly | DX: 2 |
| 128 | 2020_83 | OA | DX: 500.000 spz/mL | DX: 5 | DX: rare spermatozoa and some spermatids per tubule | DX: 3 |
| 129 | 2020_85 | TPMC<1.000.000 | DX: 200.000 spz/mL | DX: 2 | DX: germline maturation up to primary spermatocyte and spermatid stage, rare mature forms in some tubules | DX: 3 |
| **n.** | **ID** | **Diagnosis** | **TESE retrieval** | **Cryopreserved paillettes** | **Histological report** | **Histological score** |
| 130 | 2020_86 | NOA | DX: no sperm was detected (200x)  SX: 1 abnormal sperm every 50 microscopic fields was detected (200x) | DX: 0  SX: 0 | DX: tubules consisting of Sertoli cells only  SX: tubules consisting of Sertoli cells only, rare spermatids | DX: 1  SX: 2 |
| 131 | 2020_87 | NOA | SX: 1 sperm every 3 microscopic fields was detected (200x) | SX: 1 | SX: tubules consisting of germline maturation up to primary spermatocyte stage predominantly, rare spermatids in some tubules | SX: 2 |
| 132 | 2020_89 | DFI | DX: 600.000 spz/mL | DX: 6 | DX: rare spermatozoa and 7 spermatids per tubule | DX: 3 |
| 133 | 2020_93 | Anejaculation | SX: 400.000 spz/mL | SX: 4 | SX: rare spermatozoa and 10 spermatids per tubule | SX: 3 |
| 134 | 2020_95 | Retrograde Ejaculation | DX: 400.000 spz/mL | DX: 3 | DX: rare spermatozoa and some spermatids per tubule | DX: 3 |
| 135 | 2020_97 | NOA | DX: 200.000 spz/mL | DX: 2 | DX: tubules consisting of germline maturation up to primary spermatocyte stage predominantly, rare spermatids in some tubules | DX: 2 |
| 136 | 2020_101 | TPMC<1.000.000 | DX: 400.000 spz/mL | DX: 4 | DX: tubules consisting of germline maturation up to primary spermatocyte or spermatid stage, rare spermatozoa in some tubules | DX: 3 |
| 137 | 2020_104 | Anejaculation | DX: 600.000 spz/mL | DX: 4 | DX: some spermatids and rare mature forms | DX: 3 |
| 138 | 2020_105 | Criptoazoospermia | DX: 1 sperm every 10 microscopic fields was detected (200x) | DX: 1 | DX: germline maturation up to primary spermatocyte stage, extremely rare spermatids | DX: 2 |
| 139 | 2020_106 | TPMC<1.000.000 | DX: 100.000 spz/mL | DX: 4 | DX: germline maturation up to primary spermatocyte stage predominantly, rare spermatids in some tubules, mature forms almost absent | DX: 3 |
| 140 | 2020_108 | NOA | DX: no sperm was detected  SX: 1 abnormal sperm on the entire slide was detected (200x) | DX: 0  SX: 0 | DX: germline maturation up to primary spermatocyte stage predominantly  SX: germline maturation up to primary spermatocyte stage predominantly | DX: 2  SX: 2 |
| 141 | 2020_112 | NOA | DX: 500.000 spz/mL  SX: 400.000 spz/mL | DX: 2  SX: 4 | DX: rare spermatozoa and 10 spermatids per tubule  SX: some spermatozoa and 12-15 spermatids per tubule | DX: 3  SX: 3 |
| 142 | 2020_113 | OA | DX: 600.000 spz/mL | DX: 9 | DX: some spermatozoa and spermatids per tubule | DX: 3 |
| 143 | 2020_114 | OA | SX: 700.000 spz/mL | SX: 10 | SX: extremely rare spermatozoa and some spermatids per tubule | SX: 3 |
| 144 | 2020_115 | Criptoazoospermia | DX: 400.000 spz/mL | DX: 6 | DX: rare spermatozoa and 15 spermatids per tubule | DX: 3 |
| 145 | 2020_116 | TPMC<1.000.000 | DX: 400.000 spz/mL | DX: 3 | DX: some spermatids per tubule and extremely rare spermatozoa in some tubules | DX: 3 |
| 146 | 2020_119 | NOA | DX: 1 sperm every 3 microscopic fields was detected (200x)  SX: 200.000 spz/mL | DX: 2  SX: 1 | DX: tubules consisting of Sertoli cells only, rare tubules consisting of germline maturation up to primary spermatocyte or spermatid stage  SX: tubules consisting of Sertoli cells only, rare tubules consisting of germline maturation up to primary spermatocyte or spermatid stage | DX: 2  SX: 2 |
| 147 | 2020_120 | TPMC<1.000.000 | DX: 300.000 spz/mL  SX: 400.000 spz/mL | DX: 4  SX: 6 | DX: germline maturation up to spermatid stage predominantly, extremely rare mature forms  SX: germline maturation up to primary spermatocyte or spermatid stage | DX: 3  SX: 2 |
|  |  |  |  |  |  |  |
| **n.** | **ID** | **Diagnosis** | **TESE retrieval** | **Cryopreserved paillettes** | **Histological report** | **Histological score** |
| 148 | 2020_124 | Criptoazoospermia | DX: 300.000 spz/mL  SX: 300.000 spz/mL | DX: 2  SX: 3 | DX: germline maturation up to primary spermatocyte stage  SX: germline maturation up to primary spermatocyte stage, rare spermatids in rare tubules | DX: 2  SX: 2 |
| 149 | 2020_125 | TPMC<1.000.000 | DX: 300.000 spz/mL  SX: 500.000 spz/mL | DX: 2  SX: 3 | DX: some tubules consisting of germline maturation up to primary spermatocyte or spermatid stage  SX: germline maturation up to spermatid stage | DX: 2  SX: 2 |
| 150 | 2020_128 | Criptoazoospermia | DX: no sperm was detected  SX: 1 sperm every 10 microscopic fields was detected (200x) | DX: 0  SX: 1 | DX: 50% of the tubules consisting of tubular sclerosis, 50% of the tubules consisting of germline maturation up to primary spermatocyte, extremely rare spermatids  SX: germline maturation up to primary spermatocyte stage, rare spermatids | DX: 2  SX: 2 |
| 151 | 2020_129 | TPMC<1.000.000 | DX: 400.000 spz/mL  SX: 400.000 spz/mL | DX: 2  SX: 2 | DX: germline maturation up to spermatid stage, almost absent mature forms  SX: germline maturation up to spermatid stage, extremely rare mature forms in rare tubules | DX: 3  SX: 3 |
| 152 | 2020_134 | Genetic Azoospermia | DX: 1 abnormal sperm every 25 microscopic fields was detected (200x)  SX: 200.000 | DX: 0  SX: 1 | DX: tubules consisting of Sertoli cells only  SX: fibro-neuro-muscular tissue | DX: 1  SX: 1 |
| 153 | 2020_135 | Criptoazoospermia | DX: 200.000 spz/mL  SX: 200.000 spz/mL | DX: 2  SX: 2 | DX: tubules consisting of Sertoli cells only and primary spermatocytes, rare spermatids in some tubules  SX: tubules consisting of Sertoli cells only and primary spermatocytes | DX: 2  SX: 2 |
| 154 | 2020_136 | NOA | DX: 300.000 spz/mL  SX: 1 abnormal sperm on the entire slide was detected (200x) | DX: 1  SX: 0 | DX: tubules consisting of Sertoli cells only  SX: tubules consisting of Sertoli cells only | DX: 1  SX: 1 |

Criteria legend:

We assumed our assessment of ‘*Right testicle: 600.000 spermatozoa/mL (6 cryopreserved pillettes)’* to agree with the report of ‘*Right testicle: 10-12 spermatozoa per tubule’ – score: 4*.

We assumed our assessment of ‘*Right testicle: 400.000 spermatozoa/mL (5 cryopreserved pillettes)’* to agree with the report of ‘*Right testicle: 5 spermatozoa per tubule’ – score: 3*.

We assumed our assessment of ‘*Right testicle: 200.000 spermatozoa/mL (1 cryopreserved pillettes)’* to agree with the report of *Right testicle: some spermatozoa per tubule’ - score: 3*.

We assumed our assessment of ‘*Right testicle: 100.000 spermatozoa/mL (4 cryopreserved pillettes); Left testicle: no sperm was found (no cryopreserved paillette)’* to disagree with the report of ‘*Right testicle: Setoli cells only (score: 1); Left Testicle: Sertoli cells only (score: 1)’*.

We assumed our assessment of ‘*Right testicle: 1 sperm on the entire slide was found (no cryopreserved pillette); Left testicle: 1 sperm every 2 microscopic fields was found (2 cryopreserved paillettes)’* to disagree with the report of ‘*Right testicle: tubules consisting of Sertoli cells only and primary spermatocytes, spermatids in extremely rare tubules (score: 2); Left Testicle: tubules consisting of Sertoli cells only and primary spermatocytes, spermatids in extremely rare tubules (score: 2)’*.

We assumed our assessment of ‘*Right testicle: 200.000 spermatozoa/mL (2 cryopreserved pillettes); Left testicle: 300.000 spermatozoa/mL (2 cryopreserved paillettes)’* to disagree with the report of ‘*Right testicle: some spermatozoa per tubule (score: 3); Left Testicle: germline maturation up to primary spermatocyte or spermatid stage, extremely rare spermatozoa per tubule (score: 3)’*.
